# Case report: Cytokine therapy and an intracoronary autologous bone marrow-derived cell infusion with Impella support in a patient with dilated cardiomyopathy and a severely reduced ejection fraction

**DOI:** 10.3389/fcvm.2022.1002508

**Published:** 2022-09-12

**Authors:** Russell Charles Hall, Rohini Ramaseshan, Alice Reid, Daniel A. Jones, Anthony Mathur

**Affiliations:** ^1^Barts Heart Centre, Barts Health NHS Trust, London, United Kingdom; ^2^Centre for Cardiovascular Medicine and Devices, William Harvey Research Institute, Queen Mary University of London, London, United Kingdom

**Keywords:** regenerative medicine, dilated cardiomyopathy, heart failure, interventional cardiology, case report, cell therapy

## Abstract

**Introduction:**

This is the first reported case of a patient with dilated cardiomyopathy (DCM) and severely impaired left ventricular function to receive a combined treatment of granulocyte colony-stimulating factor therapy and an intracoronary delivery of autologous bone marrow-derived mononuclear cells with percutaneous circulatory assistance (the Impella CP device; Abiomed, Danvers, MA).

**Main symptoms and outcome:**

Three months post-treatment, the gentleman in his early 70s demonstrated an improvement in left ventricular ejection fraction (13–17%) and a reduction in New York Heart Association class from III to class I. There was also an improvement in his 6-minute walk test (147–357 meters), N-terminal pro-brain natriuretic peptide level (14,099–7,129 ng/l) and quality of life scores. There were no safety concerns during the treatment or follow-up.

**Conclusion:**

This case report suggests combined cell and cytokine therapy with adjunctive circulatory support could be a safe and promising treatment for patients with DCM and severely reduced ejection fraction.

## Introduction

Dilated cardiomyopathy (DCM) is a leading cause of heart failure and the most common indication for cardiac transplantation worldwide (1, 2). The prevalence of DCM is estimated at 40 cases per 100,000 individuals with an annual incidence of 7 cases per 100,000 individuals (3). The prognosis of DCM is highly variable; earlier studies reported 5-year mortality rates of 50% which have declined to 20% in more recent reports. This improvement signals both early disease detection and advances in heart failure therapy. However, despite the declining mortality rates, the prognosis and quality of life in symptomatic patients remain worse than many malignancies and serious chronic conditions such as arthritis and chronic lung disease (4).

Current conventional drug and device therapies for DCM do not correct underlying defects in cardiac muscle. Currently, heart transplantation is the only treatment option which addresses cardiomyocyte loss and fibrosis; however, the strict selection criteria and shortage of donors mean many patients do not receive a transplant. Cell therapy has generated enthusiasm as it addresses the progression of myocardial dysfunction. Autologous cell and cytokine therapy, on top of optimal medical and device therapy, has shown promise in the treatment of DCM (5–12).

REGENERATE-DCM was a phase II, randomized, placebo-controlled trial, which evaluated the efficacy of granulocyte colony-stimulating factor (G-CSF) therapy combined with an intracoronary infusion of bone marrow-derived mononuclear cells (BMMNCs) in the treatment of “no-option” dilated cardiomyopathy patients (12). The cell treated patients (*n* = 15) demonstrated a significant 5.37% increase in left ventricular ejection fraction (LVEF; mean baseline: 32.93%; 3 months: 38.30%) and significant improvements in New York Heart Association (NYHA) class (12).

Although patients with more significant left ventricular impairment appear to derive more benefit from cell therapy (13, 14), this group of patients are also at a higher risk of complications during interventional procedures, and so very few studies have exclusively targeted this patient group. Therefore, we sought to address whether the use of percutaneous circulatory support (currently only available as the Impella device) improves the safety of the procedure in patients with severely impaired left ventricular function (defined here as a LVEF of <30%). This procedure was approved by South Central - Oxford A Research Ethics Committee (REC reference: 18/SC/0195).

**Figure 1:**
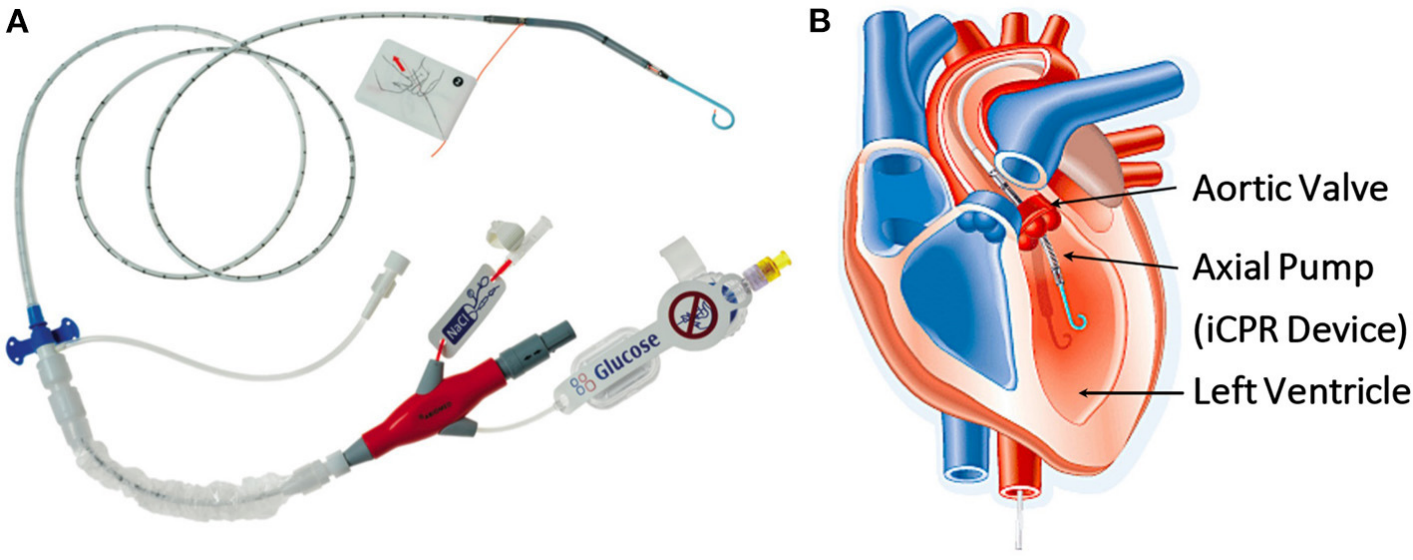
**(A)** The Impella, a minimally invasive, catheter-based circulatory support device (Abiomed, Danvers, MA). **(B)** Diagram showing the anatomical positioning of the Impella across the aortic valve. (iCPR-intravascular cardiopulmonary resuscitation). ©Abiomed.

The Impella CP device (Abiomed, Danvers, MA; Figure 1) is a percutaneous mechanical circulatory support device that can be used in clinical practice to facilitate high-risk coronary interventions (particularly in patients with severely impaired left ventricular function or cardiogenic shock) (15–17). It is known to reduce end-diastolic wall stress and pulmonary capillary wedge pressure (15, 16, 18)—thereby preventing acute decompensation. Additionally, the Impella device may improve cellular engraftment by improving coronary blood flow (19).

This is the first reported case of a patient with DCM and severely impaired left ventricular function to receive a combined treatment of G-CSF therapy and intracoronary delivery of BMMNCs with percutaneous circulatory assistance (the Impella CP device).

## Case description

### Presentation

**Figure 2:**
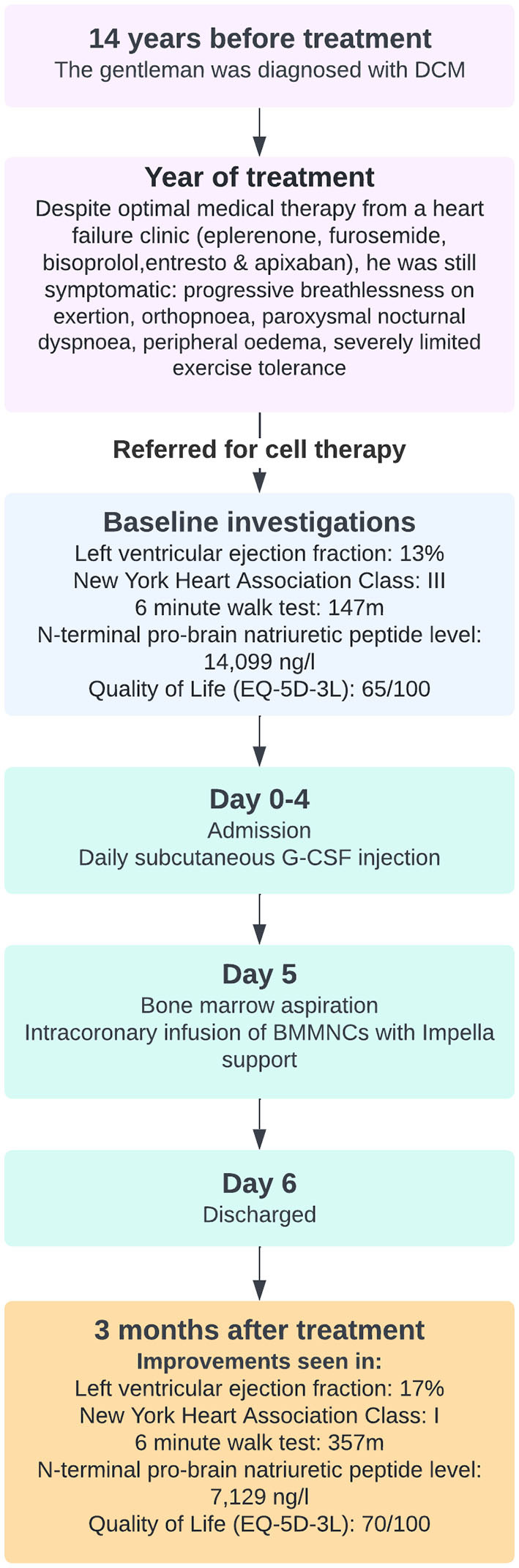
The timeline and treatment course for the patient.

Figure 2 presents the timeline and treatment course for the patient. A gentleman in his early 70s was diagnosed with DCM (presumed idiopathic) in 2008. He self-referred to our center for cell therapy having experienced a progressive increase in breathlessness on exertion, orthopnoea, paroxysmal nocturnal dyspnoea, peripheral oedema and severely limited exercise tolerance (5–10 m on flat ground) to a stage he could no longer tolerate, despite being established on maximum tolerated doses of optimal medical therapy for several years (eplerenone 25 mg OD, furosemide 40 mg BD, bisoprolol 5 mg OD, Entresto 97/103 BD, apixaban 5 mg BD). Dapagliflozin was trialed but was stopped after 10 days due to intolerance.

The patient had been advised that there were no further conventional treatment options available, hence he was interested in pursuing cell therapy. There was no direct family history of DCM (although a grandparent had cardiomegaly) and there was no other relevant past medical history. Prior to his diagnosis, he had led a very active lifestyle; but now had a severely reduced quality of life.

### Investigations

After informed consent was taken, baseline investigations were performed. The baseline transthoracic echocardiogram showed a dilated and severely impaired left ventricle with an LVEF of <30% and mild to moderate functional mitral regurgitation (vena contracta 0.39 mm). No other significant valvular abnormalities were identified. The baseline gated, rate controlled, cardiac computed tomography (CT) showed moderate left circumflex (LCx) and mid-left anterior descending (LAD) artery stenosis, and an LVEF of 13%. He had symptoms consistent with NYHA class III, Killip class II and no reported chest pains. In a 6-minute walk test he could walk 147 metres. He was noted to be atrial fibrillation with a ventricular rate of 115 beats per minute. His N-terminal pro-brain natriuretic peptide (NT Pro-BNP) was 14,099 ng/l and his high-sensitive troponin T was 18 ng/l. He performed an EQ-5D-3L quality of life questionnaire and scored his overall quality of life as 65 out of 100. Baseline observations demonstrated a body mass index of 29.5 kg/m^2^ and peripheral oxygen saturations of 97% on room air. Other observations were unremarkable.

### Treatment

The patient was electively admitted and began daily subcutaneous G-CSF (10 mcg/kg) therapy for 5 days. A progressive increase in granulocyte and total white cell counts were observed (Table 1). On day six, 54 ml of bone marrow was aspirated from the right posterior superior iliac spine under local anesthetic and sent under temperature-controlled conditions to a stem cell processing laboratory. At the laboratory, mononuclear cells were extracted from the bone marrow aspirate using a Ficoll separation technique and resuspended in 10ml of normal saline (cell infusate details are listed in Table 1). The cell infusate was then returned to the hospital cardiac catheterisation laboratory for infusion.

**Table 1:** Cell counts for peripheral blood and cell infusion.

	**Day 0**	**Day 4**	**Fold increase**
**Peripheral blood cell counts**			
White cell count (x10^9^/L)	7.9	41.5	5.3
Neutrophils (x10^9^/L)	6.3	36.6	5.8
Immature Granulocytes (x10^9^/L)	0.0	1.7	-
Monocytes (x10^9^/L)	0.5	2.6	5.2
Eosinophils (x10^9^/L)	0.0	0.1	-
Nucleated cells (x10^9^/L)	0.0	0.0	-
Lymphocytes (x10^9^/L)	1.1	2.1	1.9
**Infusion cell counts**			
Mononuclear cells (x10^6^)	164		
CD34^+^ (x10^6^)	Cell markers not measured^*^		
Endothelial progenitor cells (x10^2^)	Cell markers not measured^*^		

* Although cell surface markers are normally routinely measured, the cell processing lab was unable to do this during the pandemic.

Before cell infusion, an Impella circulatory support device was inserted under ultrasound guidance *via* the right femoral artery using a 14-F sheath as per the manufacturer's instructions. We used our existing large bore access Standard Operating Procedure based on our Transcatheter Aortic Valve Implantation (TAVI) programme (a CT scan assessment of peripheral arteries and an ultrasound-guided puncture of the femoral artery with a Manta^®^ vascular closure device).

**Figure 3:**
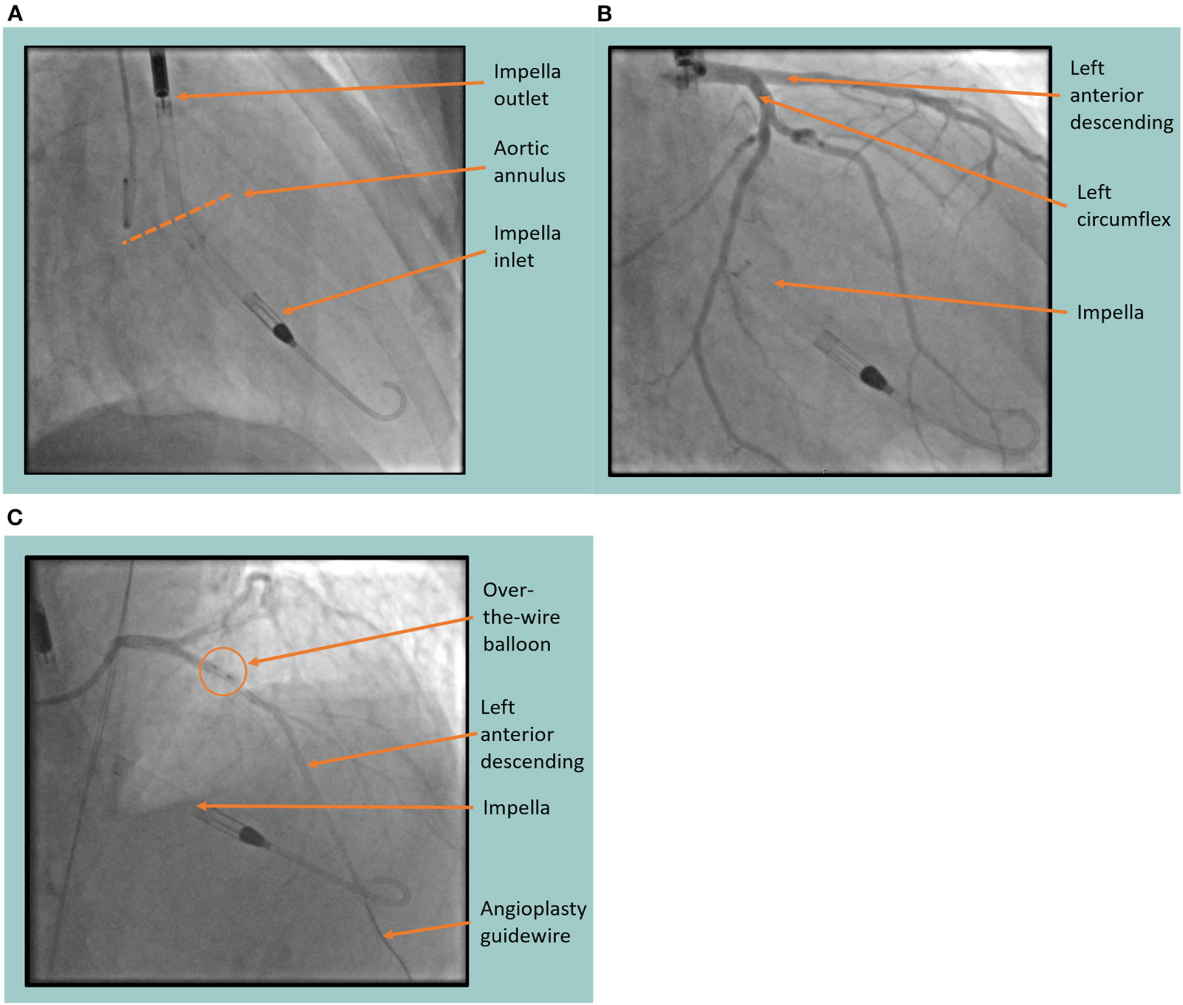
Fluoroscopic images of the Impella (Abiomed, Danvers, MA) assisted cell infusion procedure. **(A)** The Impella device is sited in the left ventricle showing the inlet and outlet port either side of the aortic annulus. **(B)** Coronary angiography is performed, identifying the left anterior descending and left circumflex coronary arteries with the Impella *in situ*. **(C)** Coronary angiogram showing the cell infusion procedure in the left anterior descending artery. Arrows indicate the position of the over-the-wire balloon in the proximal left anterior descending artery through which the cells are infused after the guide wire is removed.

**Figure 4:**
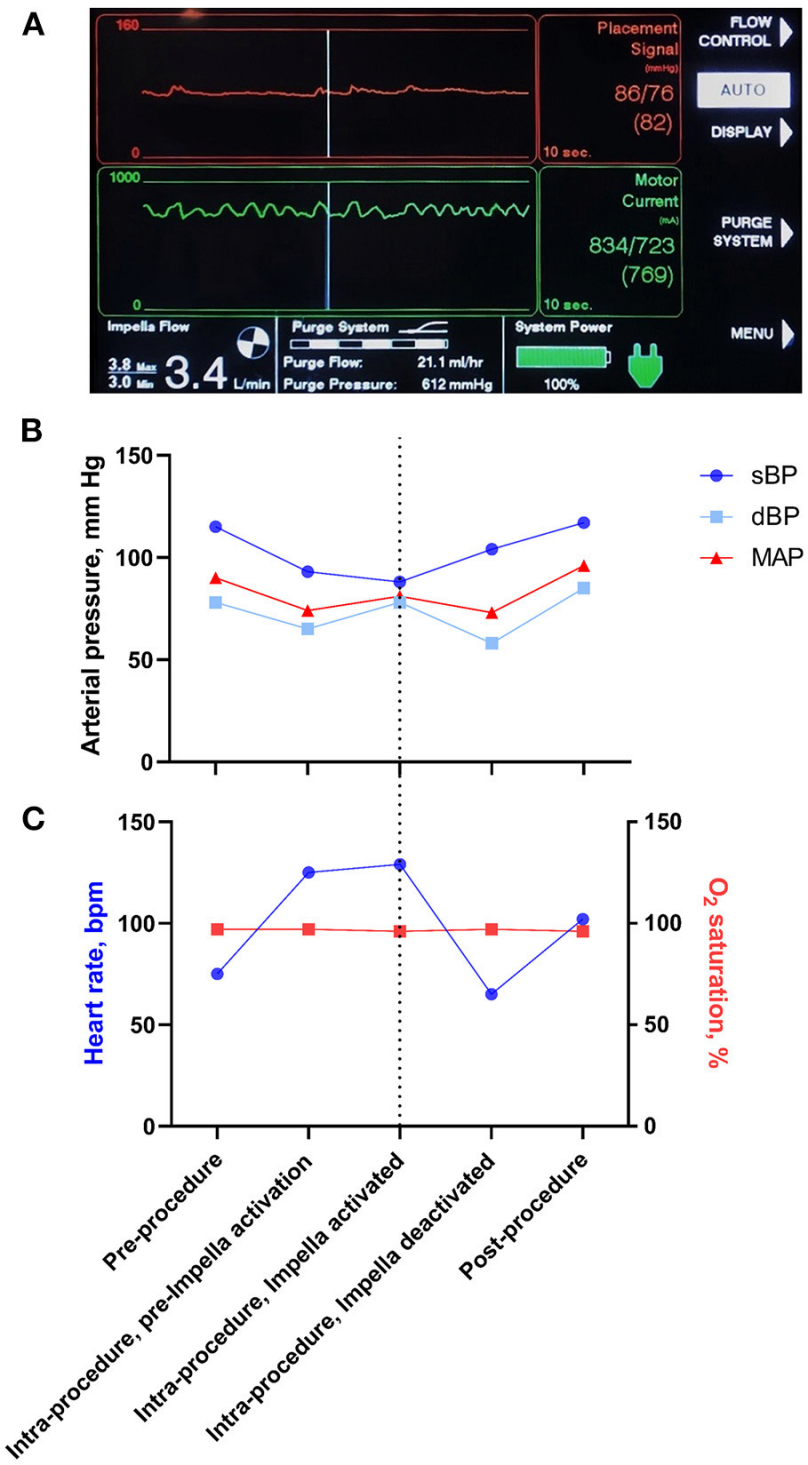
Impella read out and changes in physiological parameters during the cell infusion procedure. **(A)** Screen capture of the Impella console during the intracoronary cell infusion during a balloon occlusion of the coronary artery. The red upper panel is a measurement of blood pressure from the Impella device and demonstrates maintenance of mean arterial pressure despite a reduction in pulsatile flow. The green middle panel demonstrates the load on the Impella motor. The bottom left panel demonstrates an Impella Flow of 3.4 L/min. **(B)** Schematic representation of changes in blood pressure during the Impella-assisted cell infusion procedure. Systolic blood pressure (sBP), diastolic blood pressure (dBP) and mean arterial pressure (MAP) are shown before, during and after Impella activation. The dotted line represents the time at which cells were infused whilst the Impella was running. Note that Impella device augmented the MAP at the time of cell infusion. **(C)** Schematic representation of changes in heart rate and O_2_ saturation during the Impella-assisted cell infusion procedure. The dotted line represents the time at which cells were infused whilst the Impella was running.

Once the Impella device was sited (Figure 3A) and continuous flow was established (approximately 3.4 L/min), the BMMNCs were infused into the coronary arteries using recognized angioplasty techniques. A sheath was inserted into the radial artery and 6-F Judkins guide catheters were used to cannulate the left main and right coronary arteries consecutively (Figure 3B). For each artery (LAD, LCx, right coronary), an over-the-wire balloon (0.5 mm smaller than the vessel's diameter) was inserted over a 0.014 inch guide wire (Figure 3C). The balloon was inflated to nominal pressure to induce coronary stasis and the guide wire was removed so that cells could be infused down the central lumen of the balloon catheter. Using this technique, 3.3 ml of cells were infused over 3 minutes into each of the 3 arteries. Cardiac output was supplemented by the Impella device at 3.4 L/min to counteract the reduction in blood pressure seen on the trace when the balloons were inflated (Figure 4). Mean arterial blood pressure was maintained at 82 mmHg and the patient remained asymptomatic throughout. At the end of the 3-minute periods, the balloon was deflated, the guide wire reintroduced and a check image of the coronary artery was performed to assess for flow and any evidence of vascular trauma. No complications occurred during the procedure. Following the final infusion of cells, the flow rate on the Impella was gradually decreased before deactivation and removal. The femoral artery puncture was closed using a Manta^®^ vascular closure device. There were no significant post-procedural complications, and the patient was discharged the next day.

### Outcome

The patient returned 3 months later for follow-up. On assessment, his symptoms had improved considerably from NYHA class III to class I. On physical examination, he was clinically euvolemic, his weight had reduced from 101.2 to 87.5 kg and his Killip class had reduced from II to I. In the 6-minute walk test, he could walk a distance of 357 meters (previously 147 meters). His NT Pro-BNP had fallen from 14,099 ng/l at baseline to 7,129 ng/l. His high-sensitive troponin T had decreased from 18 mg/l at baseline to 13 mg/l. A 12-lead ECG showed atrial fibrillation at 63 beats per minute (previously 115 beats per minute at baseline). An assessment of LVEF using gated, rate control cardiac CT showed an improvement from 13 to 17%. His quality of life questionnaire (EQ-5D-3L) score had improved to 70 out of 100 (compared with 65 out of 100 at baseline). It should be noted that all these observations were taken as part of a clinic visit and, therefore, are not averaged over time.

The patient reported feeling much better since receiving cell therapy with a significant improvement in his exercise tolerance and levels of fatigue. He reported walking outdoors 2–3 times a day at a distance of approximately 400 meters without any breathlessness or fatigue (previously he could walk only 5–10 meters). His orthopnoea and paroxysmal nocturnal dyspnoea had resolved. He had managed to return to regular gardening and felt he had a “renewed interest in life.” Furthermore, at a subsequent review with his local heart failure service 7 months after cell treatment, his maintenance dose of loop diuretic (furosemide) was reduced (from 40 mg BD to 40 mg OD) as his peripheral oedema had improved.

## Discussion

Here we present the first reported case of a patient with DCM and severely impaired left ventricular function to receive a combined treatment of G-CSF therapy and an intracoronary delivery of autologous BMMNCs with percutaneous circulatory assistance (the Impella CP device). He reported a significant improvement in symptoms and quality of life which was supported by improvements in independent clinical measurements (LVEF, NT Pro-BNP, and 6-minute walk test).

Although the improvements in exercise tolerance and heart failure symptoms may be a result of weight loss and heart rate control, the independent clinical measurements suggest that these improvements are due to an overall increase in cardiac function (increased LVEF in the context of a decreased NT Pro-BNP). As the patient had been established on optimal medical therapy pre-cell treatment, this suggests that the improvements seen at 3 months appear to be linked, at least in part, to the combined G-CSF and autologous cell treatment.

Whilst trials that have used cell therapy alone have shown mixed results, trials that have used a combination of cell and cytokine therapy for the treatment of DCM have shown more consistently positive outcomes (6–12). G-CSF is a haematopoietic cytokine that not only has direct effects on the heart [cardiac regeneration (20), an accelerated healing process (21), myocardial protection from apoptosis (22) and a reduction of myocardial fibrosis (23)] but also leads to bone marrow cell maturation and an increased release, thereby exposing the heart to very high levels of circulating cells [more than by direct infusion alone (24)]. Thus, although this report is limited by being the only case of its kind to date, it is in keeping with other reported improvements from cell and cytokine therapy for DCM (albeit in patient populations with higher ejection fractions) (6–12).

Given the high complication rate associated with Impella use (25), its safety and efficacy as an adjunctive support for cell therapy in patients with poor cardiac function (LVEF <30%) needs to be established. We used our experience of large bore access and the Standard Operating Procedure based on our TAVI programme to minimize the vascular complications that have previously been associated with Impella insertion (25). In this case report, our patient did not suffer any complications from the use of the Impella and has benefitted from the procedure. Previously we have seen dips in blood pressure during cell infusion; however, during this procedure supported by the Impella device, the patient's blood pressure remained stable, and he was comfortable throughout. Overall, the procedure was very well tolerated, and the patient reported a good experience. The encouraging results from this novel use of circulatory support and cell therapy warrant further investigation in a larger patient cohort. It should be noted that patients with peripheral vascular disease or left ventricular thrombus may not be suitable candidates for Impella-supported cell infusion procedures.

## Conclusion

Therefore, as demonstrated by this case report, combined cell and cytokine therapy with adjunctive circulatory support appears to be a safe and promising treatment for patients with DCM and a severely reduced LVEF. The Impella device may be able to support cell therapy in patients with poor left ventricular function, and experience of large bore access and closure techniques may improve the vascular complication rates associated with its use.

## Patient's perspective

What wonderful treatment I received from all the staff at Barts—nothing was too much trouble for them. I still have to pinch myself to remind myself that my treatment actually happened. Such care and compassion was shown to me by all the stem cell team, and all the other members of the team of helpers whose names I do not know! Again a massive thanks to you all.

## Data availability statement

The original contributions presented in the study are included in the article/supplementary material, further inquiries can be directed to the corresponding author.

## Ethics statement

This study was reviewed and approved by the South Central-Oxford a Research Ethics Committee (REC reference: 18/SC/0195). Written informed consent was obtained from all participants for their participation in this study. Written informed consent was obtained from the participant/s for the publication of this case report.

## Author contributions

AM conceived and designed treatment. RH wrote first draft of manuscript. RR, AR, DJ, and AM wrote sections of the manuscript. All authors contributed to manuscript revision, read, and approved the submitted version.

## Funding

This work was supported by a grant awarded by the Barts Charity Cardiovascular Programme (MGU0284).

## Conflict of interest

Author AM is a Board Member of the Heart Cells Company. The remaining authors declare that the research was conducted in the absence of any commercial or financial relationships that could be construed as a potential conflict of interest.

## Publisher's note

All claims expressed in this article are solely those of the authors and do not necessarily represent those of their affiliated organizations, or those of the publisher, the editors and the reviewers. Any product that may be evaluated in this article, or claim that may be made by its manufacturer, is not guaranteed or endorsed by the publisher.

## References

[1] Harlan WR. Prevalence and etiology of idiopathic dilated cardiomyopathy (summary of a National Heart, Lung, and Blood Institute Workshop). Am J Cardiol. (1992) 69:1458–66. 10.1016/0002-9149(92)90901-A 1590237

[2] Hertz MI Aurora P Christie JD Dobbels F Edwards LB Kirk R. Scientific registry of the international society for heart and lung transplantation: introduction to the 2009 annual reports. J Hear Lung Transplant. (2009) 28:989–92. 10.1016/j.healun.2009.08.005 19782281

[3] Weintraub RG Semsarian C Macdonald P. Dilated cardiomyopathy. Lancet. (2017) 390:400–14. 10.1016/S0140-6736(16)31713-5 28190577

[4] Hobbs FDR Kenkre JE Roalfe AK Davis RC Hare R Davies MK. Impact of heart failure and left ventricular systolic dysfunction on quality of life: a cross-sectional study comparing common chronic cardiac and medical disorders and a representative adult population. Eur Heart J. (2002) 23:1867–76. 10.1053/euhj.2002.3255 12445536

[5] Reid A Mathur A. Cell-based regenerative therapy. In: The PCR-EAPCI Textbook, Toulouse (2021).

[6] Vrtovec B Poglajen G Sever M Leziac L Domanovic D Cernelc P. Effects of intracoronary stem cell transplantation in patients with dilated cardiomyopathy. J Card Fail. (2011) 17:272–81. 10.1016/j.cardfail.2010.11.007 21440864

[7] Vrtovec B Poglajen G Lezaic L Sever M Domanovic D Cernelc P. Effects of intracoronary CD34+ stem cell transplantation in nonischemic dilated cardiomyopathy patients: 5-year follow-up. Circ Res. (2013) 112:165–73. 10.1161/CIRCRESAHA.112.276519 23065358

[8] Lezaic L Socan A Poglajen G Peitl PK Sever M Cukjati M. Intracoronary transplantation of CD34+ cells is associated with improved myocardial perfusion in patients with nonischemic dilated cardiomyopathy. J Card Fail. (2015) 21:145–52. 10.1016/j.cardfail.2014.11.005 25459687

[9] Vrtovec B Sever M Jensterle M Poglajen G Janez A Kravos N. Efficacy of CD34 + stem cell therapy in nonischemic dilated cardiomyopathy is absent in patients with diabetes but preserved in patients with insulin resistance. Stem Cells Transl Med. (2016) 5:632–8. 10.5966/sctm.2015-0172 27025690 PMC4835245

[10] Vrtovec B Poglajen G Sever M Zemljic G Frljak S Cerar A. Effects of repetitive transendocardial CD34+ cell transplantation in patients with nonischemic dilated cardiomyopathy. Circ Res. (2018) 123:389–96. 10.1161/CIRCRESAHA.117.312170 29880546

[11] Bocchi EA Bacal F Guimarães G Mendroni A Mocelin A Esteves Filho A. Granulocyte-colony stimulating factor or granulocyte-colony stimulating factor associated to stem cell intracoronary infusion effects in non-ischemic refractory heart failure. Int J Cardiol. (2010) 138:94–7. 10.1016/j.ijcard.2008.06.002 18675477

[12] Hamshere S Arnous S Chowdhury T Choudry F Mozid A Yeo C. Randomized trial of combination cytokine and adult autologous bone marrow progenitor cell administration in patients with non-ischaemic dilated cardiomyopathy: the REGENERATE-DCM clinical trial. Eur Heart J. (2015) 36:3061–9. 10.1093/eurheartj/ehv390 26333366 PMC4654774

[13] Fisher SA Doree C Mathur A Taggart DP Martin-Rendon E. Stem cell therapy for chronic ischaemic heart disease and congestive heart failure. Cochrane Database Syst Rev. (2016) 12:CD007888. 10.1002/14651858.CD007888.pub3 28012165 PMC6463978

[14] Gyöngyösi M Pokushalov E Romanov A Perin E Hare JM Kastrup J. Meta-analysis of percutaneous endomyocardial cell therapy in patients with ischemic heart failure by combination of individual patient data (IPD) of ACCRUE and publication-based aggregate data. J Clin Med. (2022) 11:3205. 10.3390/jcm11113205 35683592 PMC9181462

[15] Remmelink M Sjauw KD Henriques JP de Winter RJ Koch KT van der Schaaf RJ. Effects of left ventricular unloading by Impella recover LP2.5 on coronary hemodynamics. Catheter Cardiovasc Interv. (2007) 70:532–7. 10.1002/ccd.21160 17896398

[16] O'Neill WW Kleiman NS Moses J Henriques JPS Dixon S Massaro J. A prospective, randomized clinical trial of hemodynamic support with Impella 2.5 versus intra-aortic balloon pump in patients undergoing high-risk percutaneous coronary intervention: the PROTECT II study. Circulation. (2012) 126:1717–27. 10.1161/CIRCULATIONAHA.112.098194 22935569

[17] Moura-Ferreira S Ladeiras-Lopes R MBala D Rodrigues A Braga P Gama V. The role of Impella in high-risk percutaneous coronary intervention. Rev Port Cardiol (Engl Ed). (2018) 37:623.e1–623.e4. 10.1016/j.repc.2017.05.013 29807675

[18] Meyns B Dens J Sergeant P Herijgers P Daenen W Flameng W. Initial experiences with the Impella device in patients with cardiogenic shock - Impella support for cardiogenic shock. Thorac Cardiovasc Surg. (2003) 51:312–7. 10.1055/s-2003-45422 14669126

[19] Hussain MA Colicchia M Veerapen J Weeraman D Podaru M-N Jones D. Circulatory support and stem cell therapy in the management of advanced heart failure: a concise review of available evidence. Regen Med. (2019) 14:585–93. 10.2217/rme-2018-0121 31115248

[20] Orlic D Kajstura J Chimenti S Limana F Jakoniuk I Quaini F. Mobilized bone marrow cells repair the infarcted heart, improving function and survival. Proc Natl Acad Sci USA. (2001) 98:10344–9. 10.1073/pnas.181177898 11504914 PMC56963

[21] Minatoguchi S Takemura G Chen XH Wang N Uno Y Koda M. Acceleration of the healing process and myocardial regeneration may be important as a mechanism of improvement of cardiac function and remodelling by postinfarction granulocyte colony-stimulating factor treatment. Circulation. (2004) 109:2572–80. 10.1161/01.CIR.0000129770.93985.3E 15123535

[22] Harada M Qin Y Takano H Minamino T Zou Y Toko H. G-CSF prevents cardiac remodelling after myocardial infarction by activating the Jak-Stat pathway in cardiomyocytes. Nat Med. (2005) 11:305–11. 10.1038/nm1199 15723072

[23] Li Y Takemura G Okada H Miyata S Esaki M Maruyama R. Treatment with granulocyte colony-stimulating factor ameliorates chronic heart failure. Lab Investig. (2006) 86:32–44. 10.1038/labinvest.3700367 16304579

[24] Mozid AM Jones D Arnous S Saunders N Wragg A Martin J. The effects of age, disease state, and granulocyte colony-stimulating factor on progenitor cell count and function in patients undergoing cell therapy for cardiac disease. Stem Cells Dev. (2013) 22:216–23. 10.1089/scd.2012.0139 22834565

[25] Philipson DJ Cohen DJ Fonarow GC Ziaeian B. Analysis of adverse events related to Impella usage (from the manufacturer and user facility device experience and national inpatient sample databases). Am J Cardiol. (2021) 140:91–4. 10.1016/j.amjcard.2020.10.056 33147430 PMC7796940

